# Neuropsychiatric Symptoms by Cognitive Status for Mexican-Americans Aged 85 and Older

**DOI:** 10.1093/geroni/igaa057.3293

**Published:** 2020-12-16

**Authors:** Lan Vu

**Affiliations:** University of Texas Medical Branch at Galveston, Galveston, Texas, United States

## Abstract

Older adults with dementia and cognitive impairment often experience neuropsychiatric symptoms (NPS). Few studies have investigated the presence of NPS among older Mexican-American adults. Our objective was to describe the NPS of Mexican-Americans 85 years and older according to cognitive status. Data came from wave 9 (conducted in 2016) of the Hispanic Established Populations for the Epidemiological Study of the Elderly. The final sample consisted of 381 care recipients aged 85 years and older, along with their caregivers. The 12-item Neuropsychiatric Inventory (NPI-12) was administered to measure behavioral and psychiatric symptoms among the care recipients. Cognitive impairment was defined as a score of 18 or less on the Mini Mental Status Exam (MMSE). Care recipients with a diagnosis of dementia as reported by the caregiver were also classified as cognitively impaired. Overall, 259 (68.0%) participants had one or more NPS. Logistic regression models were used to estimate the average marginal effect (range = -1 to 1) of cognitive impairment on NPS, controlling for care-recipient characteristics. Approximately 87% of care recipients with cognitive impairment had at least one NPS compared to 55.8% of those without cognitive impairment (p<0.01). The predicted probability of having one or more NPI symptoms was 0.25 percentage points (95% CI=0.14-0.35) higher for participants with cognitive impairment than those without. NPS are present in the majority of older Mexican American adults, particularly in those with cognitive impairment. Future research could also investigate sociodemographic correlates of NPS.

